# The Impact of Global Dynamic Capabilities on Governance Structure Choice of Partnership: The Moderating Effect of Ambidexterity

**DOI:** 10.3389/fpsyg.2021.619334

**Published:** 2021-03-11

**Authors:** Guoying Ren, Michael Yao-Ping Peng

**Affiliations:** ^1^Business School, Beijing Normal University, Beijing, China; ^2^School of Economics & Management, Foshan University, Foshan, China

**Keywords:** global dynamic capabilities, Governance structure, learning orientation, market orientation, international marketing

## Abstract

Research on multinational inter-organizational relationships has demonstrated that the capabilities of small and medium sized enterprises (SMEs) can be developed via partnerships, but at present, we lack studies that relate the development of such capabilities to the management of business governance structure. This study provides a new perspective on internationalized SME marketing strategies in the global context. Using a dynamic capability view of firms, the study develops hierarchical regression models linking global dynamic capabilities and governance structure. This study empirically verifies the research framework from 206 internationalized SME Taiwanese firms. The results confirm previous studies that indicate positive correlations between market orientation, learning orientation, and global dynamic capabilities. The results also indicate that the development of global dynamic capabilities impacts the choice of governance structure in firms. Our study suggests that internationalized SMEs strategically manage their autonomy and strategic options by choosing combinations of different relationship types while they decide to develop global marketing capabilities and global design capability, or both. The study also found that market orientation and learning orientation act as enabling mechanisms for building global dynamic capabilities.

## Introduction

Issues of inter-firm relationships and governance mechanisms have received considerable attention in literature on marketing. However, since most studies emphasize the effect of inter-firm governance mechanisms on resource allocation or firm performance, they are of little help in the study of how governance mechanisms are connected to the development of a firms' capabilities (Mota and de Castro, 2005), especially in an international inter-firm relationship. Most previous related studies have been made with the assistance of inter-organizational relationships on organizational capabilities but were limited by resource constraints. Internationalized SMEs cannot get access to abundant resources as large firms do to maintain the relationship with foreign firms and capitalize on the relationship. As a result, the relationship-capability-performance inference method does not apply to SMEs, especially those engaged in internationalization, thus it is necessary to adopt a back-stepping method to logically explore how the required capacity can be used to achieve the foreign relationship, which is also the purpose of this study.

A relationship is a mechanism through which firms continually combine existing resources and capabilities with the resources of their counterparts (Dyer and Singh, 1998; Mota and de Castro, 2005), to obtain new knowledge and develop new capabilities (Dyer and Hatch, 2006; Furlan et al., 2007). The use of the word relationship here refers to a mechanism constructed by the psychological cognition among individuals or organizations. When the relationship becomes closer, it means that there are many consensus, norms, beliefs, and values, which make individuals or organizations trust others and share information and knowledge at the psychological level. This study assumes governance, which is conceptualized as contractual and relational governance, to exhibit how SMEs align the composition of their governance mechanisms with the development of their capabilities.

Departing from the dynamic capability of firms, our study explores which organizational capabilities impact small and medium size enterprises (SMEs) choices in terms of governance with their foreign partners (Knight and Cavusgil, 2004; Jantunen et al., 2005). Those firms with higher dynamic capability can reconfigure their processes and structures, and thereby acquire valuable resources while differentiating product mix and marketing activities from competitors (Teece et al., 1997; Eisenhardt and Martin, 2000; Jantunen et al., 2005; Ho and Tsai, 2006; O'Cass and Weerawardena, 2010). Particularly, the emergence of the knowledge economy, intense global competition, and considerable technological advances make dynamic capability increasingly important for international competitiveness. A better understanding of the origins of capability in a dynamic international context is required (Özsomer and Simonin, 2004; Chen and Hsu, 2009). Furthermore, since the choice of governance mechanism is determined by top managers, the better they understand the capabilities of the enterprise, the more they can make a correct judgment about the governance pattern.

During the process of decision-making, the psychological cognition of top managers may be affected by the performance of the company in terms of global dynamic capability and mastery of the international market. This study thus proposes a corresponding governance pattern. To integrate the concept of dynamic capability with international marketing, this study proposes global dynamic capability (GDC) and defines it as internationalized firms processes with essential flexibility and efficacy for maintaining the value of existing global customers, creating value-adding products and global market niches in response to the changing global environment. This study aims to explore the effect of GDC on a firm's governance choices.

In addition to addressing the question of how GDCs affects the governance of SMEs, the study aims to fill another principal gap in literature research by resolving the primary question of how to develop GDCs for an internationalized SME. Zollo and Winter (Zollo and Winter, 2002) outline that the efficiency of supporting mechanisms for capability establishment are occasional incidents attributed to the selective assignment of organizational tasks. Yet, existing empirical research focuses on driving mechanisms that promote the GDcs of internationalized SMEs, such as market (Hooley et al., 2005; Hult and Ketchen, 2005; Ho and Tsai, 2006) and/or learning orientation (Baker and Sinkula, 1999). Market orientation and learning orientations are systems and values followed by internal members within the organization at a cultural level (Baker and Sinkula, 1999). Organization members gradually establish values and norms toward market culture and learning culture after being unconsciously influenced by the process of socialization at the level of psychological cognition. Internal members of the organization identify with the organizational culture in terms of its intrinsic psychological motivations and have the same tacit understanding between each other, further strengthening the spread and internalization of internal information, and improving the important capabilities of the organization. In line with the path-dependent nature of capabilities (Schreyögg and Kliesch-Eberl, 2007; Wang and Ahmed, 2007), examining the effect of organizational mechanisms (market orientation and learning orientation, namely MO and LO) on GDCs in terms of foreign market advancement and the expansion of the internationalization of SMEs has a great significance. The present study evaluates these contributions by considering how and which GDCs are established and managed.

This study contributes to existing research. First, it investigates the phenomenon of internationalization among Taiwanese SMEs. Second, it emphasizes the importance of two key GDCs (global marketing capabilities and product-design capabilities, GMCs and GPDCs) that are leveraged by SMEs when deciding specific inter-firm governance. By exploring internationalized SMEs in Taiwan, this research provides empirical evidence that GDCs significantly influence their internationalized governance structure.

## Literature Review and Research Hypotheses

### Global Dynamic Capabilities

According to Eisenhardt and Martin (Eisenhardt and Martin, 2000), dynamic capability refers to “the process of resource utilization by firms, particularly process in which firms embark on the integration, reconfiguration, acquisition and release of resources, so as to match and make changes in market” and “organizational and strategic routines based on which firms engage in new resources acquisition and allocation with the emergence, collision, separation, evolvement and extinction of markets” (p. 1107). Firms frequently undertake integration, reallocation, renewal, and recreation of resources and capabilities (Morgan et al., 2003; Schreyögg and Kliesch-Eberl, 2007) based on these processes, and what is the most important is that firms upgrade and reconstruct their core capabilities when faced with change, to keep their competitive advantages (Wang and Ahmed, 2007; Hsu and Wang, 2012).

Cadogan et al. (2002) believed that local and global differences make a significant difference to firm performance. In such a case, few studies have proposed that firms participate in competition by relying on their dynamic capabilities in foreign markets (Prange and Verdier, 2011; Pinho and Prange, 2016). This could be an option for internationalized enterprises that face rapidly changing surroundings accompanied by drastic international competition (Teece, 2007). Thus, globally reviewing the structure and operation of dynamic capability will contribute to constructing DC theory and developing more management-related opinions (Zhang and Chen, 2013).

Under the background of internationalization, Prange and Verdier (2011) define dynamic capability as a firm's engagement in the acquisition, improvement, and change of knowledge and routines in the development of capabilities, so as to affect the success of internationalization. Focus on the creation, implementation, delivery of foreign customers, and market value helps us to distinguish GDCs from dynamic capabilities (Fang and Zou, 2009). Major characteristics are indicated by these abilities: (1) systematically developing a global consistency while being conscious of particular characteristics in each country to promote the strategy customization of a country (Chen and Hsu, 2009); and (2) placing internal and external assets into adaption, integration, and reallocation to greet with opportunities in worldwide markets (Teece et al., 1997; Eisenhardt and Martin, 2000; Chen and Jaw, 2009). The survival and development of SMEs in overseas market are critically dependent on these efforts and capabilities (Abimbola and Vallaster, 2007; Chen and Hsu, 2009; Prange and Verdier, 2011).

GDC is also a high-order construct that includes various core capabilities (Morgan et al., 2003; Winter, 2003; Knight and Cavusgil, 2004; Jantunen et al., 2005; Zahra et al., 2006), as Chiarvesio et al. (2004) argue that globalization and the widespread diffusion of ICTs led traditional SMEs to develop design and marketing capabilities that they were not used to fostering. Therefore, the present study focuses on the capabilities of marketing (Coviello et al., 2000; O'Dwyer et al., 2009) and design (Cantamessa, 1999; Knight and Cavusgil, 2004; Swan et al., 2005; Abimbola and Vallaster, 2007). The study refers to suggestions from Prange and Verdier (2011); Hsu and Wang (2012), and Zhang and Chen (2013) by classifying GDCs into GMCs and GPDCs on account of the situation that better marketing and design capabilities can assist firms in dealing with the increasingly complex needs of customers in global markets or predicting rapidly changing technological advancement (Teece et al., 1997; Schreyögg and Kliesch-Eberl, 2007).

### Relationship Between Global Dynamic Capabilities and Governance Mechanisms

#### Global Marketing Capabilities (GMCs) and Governance Mechanism

Global marketing capability is often regarded as a capacity involved in the coordination and integration of internal resources and skills in rapid response to changing foreign markets or customer needs (Coviello et al., 2000). Morgan et al. (2009) found that value-creation mechanisms of marketing capability are especially immobile and hard to replicate, and most of them are irreplaceable, which was empirically verified (Vorhies and Morgan, 2005). Synthesizing insights in literature, Vorhies and Morgan (2005) distinguished the contribution of marketing capabilities to business performance, including market information management, marketing implementation, selling, pricing, channel management, marketing communication, marketing planning, and product management (Morgan et al., 2009). Since implementing lower-level capabilities is a necessary condition for developing higher-level capabilities, we focus on four marketing mix capabilities based on marketing's 4Ps: pricing capability, product management capability, place (distribution) capability, and promotion (marketing communication) capability, which are thoroughly discussed in existing literature (Vorhies and Morgan, 2005). The 4Ps are an outstanding concept relevant to countries at different stages of development or cultural property expression (Özsomer and Simonin, 2004).

Developing marketing capabilities strengthens the ability of firms to seek and select customers for SMEs that are cooperative. The capabilities expand their market beyond its local scope. SMEs based on marketing tend to emphasize the exploitation of old certainties (March, 1991) on a wider market rather than exploring new possibilities in products and patterns of interacting with customers (Furlan et al., 2007). When the SME exhibits a higher level of marketing capability, it is likely to turn to contractual governance, for it is usually unfamiliar with international transactions, especially the conditions in local markets. Since the SME has insufficient confidence in market operations in other countries and cooperation with international firms, it is well-positioned to anticipate circumstances, with contractual governance mechanism selection for dealing with such scenarios. Thus, an internationalized SME tends to prefer a legal governance structure rather than uncertainty. An SME can also depend on its ability and experience to collaborate with foreign firms to execute contract terms. Based upon the above statements, this study proposes the following hypotheses:

H1. As SMEs Develop GMCs, Contractual Governance Becomes More Required.

#### Global Product-Design Capabilities (GPDCs) and Governance Mechanism

Product development and design are significant functions in developing and utilizing firm knowledge, especially in global competition (Slater and Narver, 1998). As a consequence, robust design capabilities provide more potential when developing products that are acceptable to more extensive targeted segments, events, and/or conditions and enable costs to be offset by anticipating organizational benefits (Cantamessa, 1999). As time goes on, the quantity of new components, parts, materials, and technologies across a product family decreased by achieving robust design. This means increasing the variety of product lines, keeping down the cost of manufacturing, accelerating the technological advancement of products, and facilitating new product marketing, thus resulting in a subsequent increase of the number and scale of target segments (Rindova and Petkova, 2007).

Broadly speaking, GPDCs spring up, which are involved in the creation of products, processes, and knowledge architectures that are robust across utilization, technology vicissitudes, and contextual differences. Swan et al. (2005) came up with four kinds of capabilities that are conducive to firm performance: (1) functional; (2) aesthetic; (3) technological; and (4) quality. Each functional domain is equipped with potential robust capability, no matter alone or in connection with others. Based on the definition, the breadth capability of a robust functional product contains product design with similar technologies but with versatility or adaptability which is extended to a significant family of variants concurrently available or easily modifiable for domestic and international uses. Robust aesthetic product capability needs products that are equipped with visual information and gratified in domestic and/or diversified foreign markets. Robust technology capability is composed of the selection of core product technologies and materials which meet the technical and customer needs of not only present product generations but also future product generations. Robust capacity based on quality involves addressing problems during the design process (Kaynak, 2003) that take initiative and exclude deviations from requirements that have been built in diversified surroundings (manufacturing, assembly, and customer service conditions) (Swan et al., 2005).

Design capabilities also facilitate the absorptive capacity of SMEs, which accelerates learning from customers and creates relevant rents that originate from reciprocal commitment (Dyer and Singh, 1998; Ploetner and Ehret, 2006). SMEs' closer relationships with other firms that are not necessarily situated in the original local cluster can be achieved by investment in design capabilities. SMEs based on design adopt design capabilities to open up the governance mechanism of relationships among firms and participate in more complicated and remunerative ones (ie. relational governance). After making investments in design capabilities for new knowledge channel exploration, they utilize this knowledge to modify their governance mechanism toward more autonomous allocations (Sidhu et al., 2007). This problem has been explored by Martin and Salomon (2003), who are aware of the fact that too much recessiveness can make an internationalizing SEM bear more. Their relational governance determines the success of design capability and makes it available for them to transform technological knowledge into commercially successful innovations. An SME based on design may have an interest in collaborating with large international firms that possess effective distribution channels for accessing other markets. Design-based SMEs reduce traditional relationships, they re-configure their governance mechanisms in a variety of ways that are contingent on the industry, products, and technologies, with little focus on contractual governance, the most on relational governance and others on both (Furlan et al., 2007). Our second hypothesis is:

H2. As SMEs Develop GPDCs, Relational Governance Becomes More Required.

#### The Ambidextrous Effect of Global Dynamic Capabilities on Governance Mechanism

According to March's (1991) view, developing marketing capabilities or design capabilities requires time and resource investments by the organization, indicating that it is a strategic decision to develop either type of capability or to concurrently develop both of them (Bitar and Hafsi, 2007). However, there is still a lack of understanding on the joint effect of GPDCs and GMCs. This study explores the concept of ambidexterity to explore these effects further. The concept of ambidexterity represents the combination of both GPDCs and GMCs via loosely coupled and differentiated subunits or individuals (Zhang and Chen, 2013), each of which specializes in either GPDCs or GMCs (Peng and Lin, 2019; Peng et al., 2019). However, ambidextrous organizations conduct inspections and other activities with speed and flexibility to offer new products or services in global markets (Andriopoulos and Lewis, 2009; Vahlne and Jonsson, 2017; Peng and Lin, 2019). The basic logic of GPDCs and GMCs are different and somewhat distinct. When selecting a capability to develop, firms are primarily concerned with how much investment different types of activities require. Developing GPDCs and GMCs simultaneously within the same unit not only involves scarcity of resources but also challenges the selection of governance mechanisms. However, several scholars have claimed that ambidexterity is a structural mode (Andriopoulos and Lewis, 2009; Peng and Lin, 2019; Peng et al., 2019), which enables firms to establish various organizational structures to engage in activities with contradictions and opposites through differentiation strategy. In other words, the concept of ambidexterity enriches SMEs with the driving force to develop GPDCs and GMCs simultaneously, which require both a flexible peculiarity possessed by the internal structure and cooperation from close partnerships. Thus, when the global market environment changes, GMC is not enough to help these firms achieve better performance, and GPDC is also needed. This indicates that there is an inherent ambidextrous relationship between GPDCs and GMCs, which informs our third hypothesis:

H3. As SMEs Develop Both GPDCs and GMCs, Relational Governance Become More Required.

### Development of Global Dynamic Capabilities in Internationalized SMEs

Two causal mechanisms are conducive to the progress of GDC for internationalized SMEs: MO and LO (Baker and Sinkula, 1999). With support for building capabilities, managers can create an organizational context, such as organizational structure and organizational culture, to improve the efficiency and responsiveness of integrating, combining, and deploying resources (Hult and Ketchen, 2005). Chen and Jaw (Chen and Jaw, 2009) assert that GDCs adapt, integrate, and reallocate internal and external assets when they encounter opportunities in a global environment. Scholars have indicated that MO and LO can be regarded as indispensable cultural factors during the growth of an enterprise (Narver and Slater, 1990; Sinkula et al., 1997; Baker and Sinkula, 1999), influenced by the psychology of internal members within the organization, including factors such as collective identification, collective system, and collective norm, etc. A series of support initiatives are conducted by SMEs to pinpoint the internal and external assets that are utilized, integrated, and reallocated in determining which MO concentrates on the perception of external market information (Ho and Tsai, 2006; Teece, 2007; Wang and Ahmed, 2007) and LO concentrates on internal knowledge acquisition, assimilation, transformation and application (Eisenhardt and Martin, 2000; Zollo and Winter, 2002).

#### Capability-Building Support Activity: Market Orientation

Kohli and Jaworski (1990) and Narver and Slater (1990) suggest that the actions, decisions, and attitudes of senior managers “trickle down” organizational levels to the employees that implement strategies. This study adopts the widely-used perspective from Narver and Slater. MO and thus included three components: customer orientation, competitor orientation, and inter-functional coordination. It is agreed by most authors that all three factors are crucial, which gives a comprehensive view of a firm's ability in terms of the effective collection and utilization of market information (e.g., Narver and Slater, 1990; Kohli et al., 1993).

Globalization contributes to the occurrence of customers who are better organized, better informed, and more demanding (Knight and Cavusgil, 2004). MO may be extremely significant for capability-building, providing support mechanisms through information from other organizations, creating insights into how these should be managed in response to market circumstances (Slater and Narver, 1995; Ho and Tsai, 2006). Based on the background of risk in export, Morgan et al. (2003) put forward two types of export knowledge deriving from the knowledge-based theory: market information and knowledge related to experience. The former identifies with the KBV's conceptualization of “informational” (also regarded as “declarative” or “know-what”) knowledge of customers, contenders, and channels (Morgan et al., 2009), and more extensive environmental data organized to endow meaning (Kogut and Zander, 1992). The latter conforms to the KBV's conceptualization of “experiential” (also known as “procedural” or “know-how”) knowledge concerning accumulated skills that enable the effective and efficient fulfillment of required tasks. These enhance the GMCs and GPDCs of SMEs. GMCs and GPDCs may lead to the overlap of low-level targets on account of discrepancies in business requirements, both are common in creating superior customer value and satisfaction (Kaynak, 2003; Hult and Ketchen, 2005; Jantunen et al., 2005; Menguc and Auh, 2008; Chen and Jaw, 2009). To evaluate how internationalized firms improve market performance in multiple countries, MO assumes that capability-building supporting activities integrate available informational and experiential knowledge (Baker and Sinkula, 2002; Knight and Cavusgil, 2004) in concerned GDCs as a pivotal theoretical premise. In the same way, firms in international markets may hunt for more market knowledge relating to a specific place in order to select resource combinations that enable high productivity and that make them more effective and efficient in GMCs and GPDCs (Zhang and Tansuhaj, 2007; Morgan et al., 2009) than those in local markets. Based upon the above argument, we propose the following hypotheses:

H4a. MO Is Positively Related to GMCs
H4b. MO Is Positively Related to GPDCs

#### Capability-Building Support Activity: Learning Orientation

Baker and Sinkula (1999) regard LO as a series of organizational values that concern an exporter's knowledge creation and utilization, outlining that it is a progressive learning concept (Double-Loop Learning). These values prompt organizations to perfect extant paradigms and promote paradigm transfer (Miner et al., 2001). Thereby, LO is available to augment the heterogeneity and scope of an exporter's knowledge, as well as improving organizational effectiveness. Sinkula et al. (1997) accounted for LO based on three dimensions: dedication to learning, open-mindedness, and shared vision. LO is regarded as a set of organizational values, with abilities in knowledge creation, dissemination, and utilization (Sinkula et al., 1997). It goes beyond adapting to changes in marketplaces and involves knowledge-questioning values that induce generative learning (Sinkula et al., 1997). An organizational learning culture thus manifests as a behavioral norm that has an impact on market information development and processing (Zhang and Tansuhaj, 2007).

GDCs can be acquired through LO mechanisms and mechanisms of organizational internal knowledge integration (Baker and Sinkula, 1999, 2002). According to Liu (2005), dynamic capabilities establish competitive advantages during procedures of knowledge learning. Furthermore, the comprehensive learning mechanisms of internal knowledge are conducive to dynamic capabilities and competitive advantage enhancement (Eisenhardt and Martin, 2000). Lumpkin and Lichtenstein (2005) argue that organizational learning includes improving practices and developing into new fields through new knowledge creation, new understanding establishment, misalignment detection, and correction, which may accelerate entrepreneurial efforts. As Jantunen et al. (2005) describe, value creation involves ways of recognizing entrepreneurial opportunities and forward-looking strategic orientation that are significant for the framework of dynamic capability. Since GDCs are built on the consolidation and adjustment of resources, a learning culture contributes to the shared explanation of knowledge, strives for a more efficient and faster way to develop organizational routines in an international firm (Slater and Narver, 1995), and assists in transforming accumulated resources into GMCs and GPDCs (Fang and Zou, 2009). As a consequence, internationalized SMEs possessing high LO will largely adopt learning activities for GDCs development. Hence, this study proposes the following hypotheses:

H5a. LO is positively related to GMCs.
H5b. LO is positively related to GPDCs.

**Figure 1:**
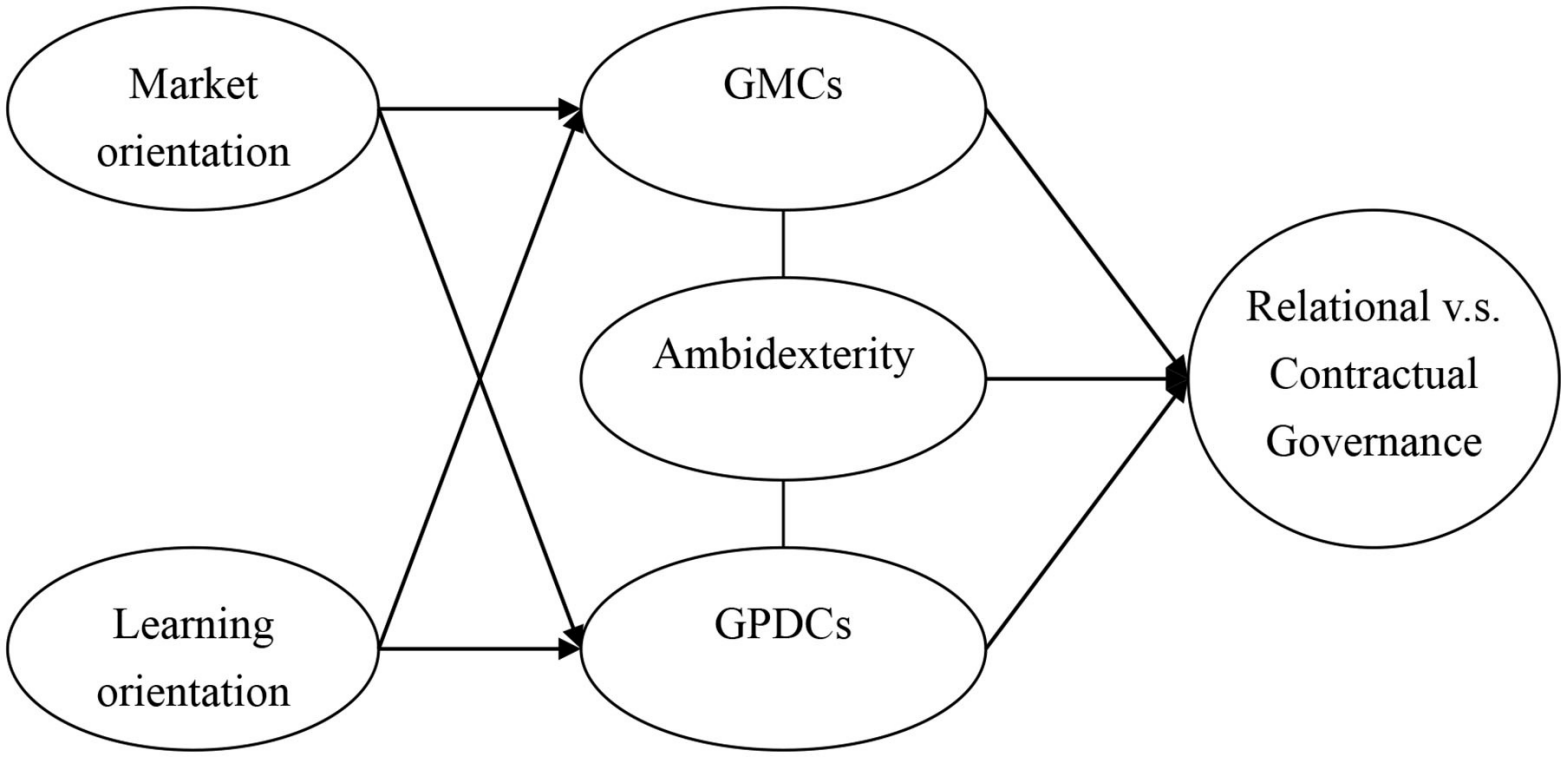
Conceptual framework.

Building on the above contributions, we thus present our conceptual GDC model of internationalized SEMs in Figure 1.

## Methodology

### Sampling and Procedures

Most empirical studies correlate dynamic capabilities with firm performance and/or the examined success (or failure) of firms in developed nations such as the US (Knight and Cavusgil, 2004; Swan et al., 2005), Australia, New Zealand (O'Cass and Weerawardena, 2010), the UK (Morgan et al., 2003), and Finland (Jantunen et al., 2005). This study used a sample of Taiwanese SMEs.

The survey of targeted SMEs, which are defined as enterprises with <500 employees, was conducted by computer-assisted telephone interviews. This study surveyed informants' (CEOs, vice presidents, senior managers) knowledge and responsibilities in companies. These top managers know the company's businesses and are familiar with actual situations of internationalization, capability development, and operation. We sent 1,000 questionnaires and received 224 completed answers, a response rate of 22.4%. After eliminating 18 invalid questionnaires, there were 206 left, with an effective response rate of 20.6%.

When self-reported questionnaires are used to collect data at the same time from the same participants, common method variance (CMV) may be a concern. A *post hoc* Harman one-factor analysis was used to test common method variance (Podsakoff and Organ, 1986). The factor analysis produced neither a single factor nor one general factor that accounted for the majority of the variance. According to the results, the test thus failed to identify that common method variance was a problem.

### Measures

#### Market Orientation

Consistent with previous marketing literature, we operationalized this construct as a higher-order construct of customer orientation (six items), competitor orientation (four items), and inter-functional coordination (five items) developed by Narver and Slater (1990).

#### Learning Orientation

Following Baker and Sinkula's (1999) study, we have defined LO as a “higher-order construct composed of commitment to learning, shared vision, and open-mindedness,” including four items for commitment to learning, five items for a shared vision, and another four items for open-mindedness.

#### Global Marketing Capability

GMCs were measured via a seven-point scale developed by O'Cass and Weerawardena (2010) to capture the capacity of firms to use marketing tools and reach target global markets effectively, by focusing on firms' capability to undertake key marketing functions in the global context.

#### Global Product-Design Capability

We adopted a conceptual framework, developed by Swan et al. (2005), and identified four dimensions for measuring GPDCs, including three items for functional capability, three items for esthetic capability, three items for technological capability, four items for Quality-based capability.

#### Ambidexterity

Of particular interest is our derived measure of ambidexterity. Following Gibson and Birkinshaw (2004), we created this construct as a multiplicative term of marketing capability and design capability. This approach is also consistent with the theoretical conceptualization of the term ambidexterity (e.g., Tushman and O'Reilly, 1996).

#### Relational Governance

Following the research of Claro et al. (2003), two dimensions constructed relational governance: joint planning (four items) and joint problem solving (four items).

#### Contractual Governance

We measured contractual governance using a formative scale taken from Ferguson et al. (2005), examining the extent to which legal ties have been implemented in the exchange, using four items that we have adapted to the global context. Above all items were shown in Table 1.

**Table 1:** Scales items factor loading.

**Second order factor**	**First order factor**	**Items**	**Loading**
Market orientation	Customer orientation	1. We closely monitor and assess our level of commitment in serving customer's needs. 2. Business strategies are driven by the goal of increasing customer value. 3. Our competitive advantage is based on understanding customer needs. 4. Our business objectives are driven by customer satisfaction. 5. We pay close attention to after-sales service. 6. We frequently measure customer satisfaction.	0.93 0.87 0.94 0.91 0.94 0.95
	Competitor orientation	7. Top management regularly discuss competitors' strength and weaknesses. 8. We respond rapidly to competitive actions. 9. Customers are targeted when we have an opportunity for competitive advantage. 10. Our salespeople share information about competitors.	0.90 0.86 0.94 0.86
	Interfunctional coordination	11. Top management regularly visits important customers 12. Information about customers is freely communicated throughout our organization. 13. Business functions within are integrated to serve the target market needs. 14. Our managers understand how employees can contribute to value of customers. 15. We share resources with other business units.	0.89 0.89 0.75 0.75 -
Learning orientation	Commitment to learning	1. The sense around here is that employee learning is an investment, not an expense. 2. The basic values of this organization include learning as key to improvement. 3. Learning in my organization is seen as a key commodity necessary to guarantee organizational survival. 4. Managers basically agree that our organization's ability to learn is the key to our competitive advantage.	0.74 0.68 0.77 0.75
	Shared vision	5. All employees are committed to the goals of the organization. 6. There is total agreement on our organizational vision across all levels, functions, and divisions. 7. There is a commonality of purpose in my organization. 8. Employees view themselves responsible for the direction of the organization. 9. Employees view themselves as partners in charting the direction of the organization.	0.73 0.66 0.67 0.72 0.63
	Open-mindedness	10. Managers agree that it is important to accept diverse viewpoints. 11. We are not afraid to reflect critically on the shared assumptions we have made about our customers. 12. Our organization pays much attention to original ideas. 13. The culture in our organization emphasizes continuous innovation.	−0.76 0.73 0.69
GMCs		1. Global sales people 2. Global distribution 3. Global promotion and advertising 4. Global market research 5. Global product differentiation 6. Global new product introduction 7. Global marketing success 8. Global marketing capability allows firm to compete	0.63 - - 0.77 0.78 0.67 0.77 0.84
GPDCs	Functional Capability	1. Spent on designing this product to be easily stretched into a family of products usable across domestic and multiple foreign market situations. 2. Spent on this capability vs. the total time spent on the other three capabilities. 3. The relative resource commitment to R&D Functional Capabilities.	0.77 0.97 0.97
	Aesthetic Capability	4. Spent on designing this product to be visually acceptable across domestic and multiple foreign market situations. 5. The relative time commitment to R&D Aesthetic Capabilities 6. The relative resource commitment to R&D Aesthetic Capabilities	0.95 0.98 0.95
	Technological Capability	7. Spent on selecting core product technologies that satisfy not only present requirements but are applicable to future product generations 8. The relative time commitment to R&D Technological Capabilities 9. The relative resource commitment to R&D Technological Capabilities	0.95 0.98 0.97
	Quality-based Capability	10. spent on solving problems in the design stage that proactively eliminate deviations from established requirements in manufacturing and assembly 11. spent on solving problems in the design stage that increase the usability and durability of the product in diverse customer usage situations	0.95 0.97
		12. The relative time commitment to R&D Quality Capabilities 13. The relative resource commitment to R&D Quality Capabilities	0.97 0.97
Relational governance	Joint planning	1. We plan volume demands for the next seasons together with this buyer. 2. We plan the new products and varieties demands for the next seasons together with this buyer. 3. This buyer provides us with sale forecasts for the products our company sells to them. 4. We share long-term plans of our products with this buyer.	0.82 0.77 0.85 0.75
	Joint problem solving	1. This buyer and we deal with problems that arise in the course of the relationship together. 2. This buyer and we do not mind owing each other favors. 3. In most aspects of the relationship with this buyer, the responsibility for getting things done is shared. 4. This buyer and we are committed to improvements that may benefit the relationship as a whole.	0.82 0.87 0.73 0.70
Contractual governance		1. Relationship governed by rules and regulations of contract 2. We would find satisfactory solution to disagreement. whether it is based on the agreement or not 3. Contract adapted to company's specific needs 4. Contract changes as client's business changes	0.75 0.83 0.90 0.44

#### Control Variables

Firm size is an important dimension of the study. The upper size boundary for a small and medium-sized firm lacks consensus, but the current study adopted a common choice, namely 499 (Hooley et al., 2005). In addition to considering the size of small and medium-sized enterprises, their capital amount, profitability, and international experiences, the control variables used by Swaminathan and Moorman (Swaminathan and Moorman, 2009) should also be taken into consideration, which includes marketing, advertising, and R&D expenditure.

## Result and Analysis

### Reliability and Validity

This study adopts confirmatory factor analysis (CFA) using AMOS 23.0 to measure and also takes into consideration the criteria of convergent validity set by Hair et al. (2010), that is, (1) All the standardized factor loadings must be >0.5 and reach the level of significance (2) the value of composite reliability (CR) must be higher than 0.7 (3) the average variance extracted (AVE) must exceed 0.5. As all the coefficients of the factor loadings of measured variables in this study are >0.5. All the measured variables are significant, so the measurement model studied has considerable convergent validity. What is more, the CR and AVE values of the variables in this study range from 0.77~0.98 and 0.53~0.93 respectively, and all the variables showed good fitness, indicating good convergent validity between the variables in this measurement mode (shown in Table 2). All three criteria for convergent validity were met, and correlation coefficients were less than the square root of the AVE within one dimension, suggesting that each dimension in this study had good discriminant validity.

**Table 2:** Scales measurement.

**Variables**	**M**	**SD**	**1**	**2**	**3**	**4**	**5**	**6**	**7**	**8**	**9**	**10**	**11**	**12**	**13**	**14**	**15**	**16**	**17**	**18**	**19**
Market orientation (α = 0.938)	5.13	0.88																			
1. Customer orientation	5.23	0.96	(0.92)																		
2. Competitor orientation	5.07	1.01	0.86^a^	(0.89)																	
3. Interfunctional coord.	5.04	0.94	0.67^a^	0.67^a^	(0.82)																
Learning orientation (α = 0.867)	5.01	0.82																			
4. Commitment to learning	4.89	1.20	0.27^a^	0.27^a^	0.31^a^	(0.74)															
5. Shared vision	5.05	0.78	0.29^a^	0.22^a^	0.40^a^	0.60^a^	(0.73)														
6. Open-mindedness	5.12	0.94	0.22^a^	0.17^b^	0.39^a^	0.45^a^	0.73^a^	(0.73)													
7. GMCs	5.34	0.87	0.73^a^	0.63^a^	0.54^a^	0.31^a^	0.48^a^	0.36^a^	(0.75)												
GPDCs (α = 0.948)	5.23	0.92																			
8. Functional Cap.	5.41	0.97	0.65^a^	0.57^a^	0.49^a^	0.39^a^	0.45^a^	0.30^a^	0.88^a^	(0.91)											
9. Aesthetic Cap.	5.27	1.06	0.67^a^	0.59^a^	0.53^a^	0.20^a^	0.30^a^	0.22^a^	0.85^a^	0.82^a^	(0.96)										
10. Technological Cap.	5.28	1.04	0.58^a^	0.70^a^	0.51^a^	0.30^a^	0.32^a^	0.22^a^	0.76^a^	0.82^a^	0.78^a^	(0.96)									
11. Quality-based Cap.	5.01	0.99	0.51^a^	0.60^a^	0.56^a^	0.51^a^	0.45^a^	0.34^a^	0.72^a^	0.78^a^	0.72^a^	0.79^a^	(0.96)								
Relational governance (α = 0.926)																					
12. Joint planning	5.41	0.88	0.75^a^	0.66^a^	0.63^a^	0.35^a^	0.40^a^	0.29^a^	0.77^a^	0.71^a^	0.68^a^	0.61^a^	0.60^a^	(0.80)							
13. Joint problem solving	5.31	0.96	0.70^a^	0.74^a^	0.62^a^	0.29^a^	0.34^a^	0.26^a^	0.75^a^	0.64^a^	0.71^a^	0.72^a^	0.60^a^	0.88^a^	(0.78)						
14. Contractual governance	4.84	1.04	0.49^a^	0.50^a^	0.70^a^	0.20^a^	0.41^a^	0.29^a^	0.47^a^	0.37^a^	0.47^a^	0.41^a^	0.42^a^	0.53^a^	0.55^a^	(0.75)					
Control variables																					
15. Firm size	3.30	1.09	−0.03	0.00	−0.06	0.01	−0.02	−0.01	−0.05	−0.04	−0.04	−0.01	0.01	−0.04	−0.05	0.07	1				
16. Organizational slack	2.59	1.44	−0.03	−0.07	−0.01	0.02	0.12	0.16	0.04	0.01	0.01	−0.01	0.05	0.07	0.01	0.04	0.46^a^	1			
17. % of marketing	0.12	0.10	0.03	0.03	0.02	0.06	−0.02	−0.06	0.03	0.09	0.01	0.01	0.08	0.03	−0.03	−0.07	0.16^b^	0.09	1		
18. % of R&D	0.14	0.11	0.02	0.06	0.05	0.06	−0.01	−0.07	−0.01	0.05	0.02	0.05	0.06	0.03	0.01	−0.02	0.06	−0.09	0.66^a^	1	
19. Internationalized	13.1	7.00	−0.12	−0.10	−0.05	0.02	0.02	0.05	−0.10	−0.13	−0.12	−0.08	−0.10	−0.06	−0.07	0.00	0.11	0.01	0.00	−0.04	1
Cronbach's α	–	–	0.914	0.823	0.811	0.939	0.536	0.610	0.838	0.875	0.885	0.820	0.821	0.867	0.851	0.811	–	–	–	–	–
CR	–	–	0.97	0.94	0.89	0.83	0.85	0.77	0.88	0.93	0.97	0.98	0.98	0.88	0.86	0.83	–	–	–	–	–
AVE	–	–	0.85	0.79	0.68	0.54	0.53	0.53	0.56	0.82	0.92	0.93	0.93	0.64	0.61	0.56	–	–	–	–	–

a *if p < 0.01*.

b *if p < 0.05*.

### The Relationship Between Global Dynamic Capabilities and Governance Structure

To investigate the relationship between internationalized SMEs' global dynamic capabilities (GMCs and GPDCs) and governance structure (relational governance and contractual governance), this study divided the sample into four subsamples. Following K-means algorithm (Hartigan and Wong, 1979), groups were constructed based on GMCs and GPDCs as low GPDCs/GMCs (lack of orientation), low GPDCs/high GMCs (GMCs oriented SMEs), high GPDCs/low GMCs (GPDCs oriented SMEs) and high GPDCs/GMCs (ambidextrous SMEs). A paired sample *t*-test was conducted on the summed mean for the relational and contractual governance, the results of which are shown in Table 3. Some significant differences were found between the relational and contractual governance in groups 2, 3, and 4. This demonstrates that GMCs-oriented SMEs will have more preference on relational governance than contractual governance that H1 is not supported.

**Table 3:** Paired sample *t*-test of relational and contractual governance.

**Category**	***N***	**Governance structure**	**Mean**	**SD**	***t***	***p-value***
Group 1	12	Relational governance	3.82	1.53	0.498	0.628
		Contractual governance	3.71	1.26		
Group 2	55	Relational governance	5.18	0.49	2.667^**^	0.010
		Contractual governance	4.77	0.90		
Group 3	36	Relational governance	4.61	0.55	3.682^***^	0.001
		Contractual governance	4.13	0.78		
Group 4	103	Relational governance	5.90	0.57	7.863^***^	0.000
		Contractual governance	5.26	0.93		

** *p < 0.01*.

*** *p < 0.001*.

If GPDCs-oriented SMEs cooperate with foreign partners by contract as they develop a foreign market, face the product demand of various customers, and devote themselves to GPDCs, they will not be able to acquire effective information about the products on the market. Relational governance, joint planning and joint problem-solving will be conducive to promoting interaction between manufacturers and their foreign partners, as they are willing to provide complete and accurate information and exchange it with each other, so manufacturers can combine this information with internal existing products efficiently and advance their GPDCs. Thus, H2 gains support. Finally, to test the ambidextrous effect of developing GMCs and GPDCs together, we found a significant difference between the groups, indicating that ambidextrous SMEs prefer relational governance over contractual governance. Thus, H3 was supported.

### The Impact of Capability-Building Support Activity on Global Dynamic Capabilities

The hypothesized relationships were tested based on hierarchical multiple regression analysis. Hierarchical regression enables analysis of the proportion of variance that is shared exclusively with each additional variable. As shown in Table 4, all proposed relationships are significant. The coefficient on the relationships from the market orientation to GMCs and from the market orientation to GPDCs are.631 (*p* < 0.01) and.611 (*p* < 0.01) respectively. These positive relationships suggest that H4a and H4b are supported.

**Table 4:** Tests for the impact of capability-building support activity on global dynamic capabilities.

	**Dependent variables**			
	**GMCs**		**GPDCs**	
	**Model 1**	**Model 2**	**Model 1**	**Model 2**
**Main Effects**				
Market orientation		0.631^***^		0.611^***^
Learning orientation		0.215^***^		0.223^***^
**Control Variables**				
Firm size	−0.074	−0.051	−0.030	−0.007
Organizational slack	0.068	0.057	0.031	0.018
Expenditures of marketing	0.065	0.058	0.044	0.038
Expenditures of R&D	−0.043	−0.066	0.020	−0.003
Internationalized experience	−0.099	−0.042	−0.110	−0.056
**Overall Model**				
*R^2^* (adj. *R^2^*)	0.019 (0.006)	0.556 (0.540)	0.017 (0.007)	0.533 (0.517)
*F*-statistic	0.768	35.377^***^	0.695	32.336^***^

*** *p < 0.001*.

In H5a and H5b, we predicted that the learning orientation has supported the development of global dynamic capabilities in all two dimensions: GMCs and GPDCs. As shown in Table 4, we have found that learning orientation positively influences both GMCs (β = 0.215, *p* < 0.001) and GDCs (β = 0.223, *p* < 0.001). These positive relationships suggest that H5a and H5b are supported.

## Discussion and Implications

A major area of contribution to the literature is the evaluation of alternative global dynamic capabilities as determinants of the governance structure. Two key global dynamic capabilities, GMCs and GPDCs, are the central players in explaining a firm's selection of governance structure. Both global dynamic capabilities seem to be core to how marketing works, but no previous studies have confirmed their concurrent contribution in the international context.

Two capability-building support mechanisms, MO and LO, support the two key global dynamic capabilities. This finding is broadly consistent with Hooley et al. (2005), except they do not support H5. MO has a strong influence on GMCs and, as expected, a stronger influence on GPDCs. This part also works in concert with previous studies, indicating that superior and deeply rooted organizational culture is conducive to improving communication and psychological distance among members within the organization (Peng and Lin, 2017; Peng, 2020), strengthening collaboration opportunities between each other and improving the GMCs and GPDCs required by enterprises for leverage application in the international market. Moreover, according to the viewpoints on resource management of Sirmon et al. (2007), organizations should use various processes to realize resource combinations, including acquiring, accumulating, and divesting. The learning orientation discussed in this research means creating competitive advantages or intangible assets, which are used to add value for enterprises as their employees acquire knowledge. This concept encourages people to develop and accumulate resources in the internal organization, which happens to coincide with Sirmon et al. (2007) in terms of “accumulating.” Thereinto, MO and LO are two kinds of organizational culture, as ambidextrous culture proposed by Liu et al. (2019) that not only motivates employees to act based on their intrinsic psychological needs such as autonomy, competence, and relatedness, but also further promotes the overall active involvement of organizational members in the development, communication, dissemination, and implementation of organizational goals.

Additionally, the so-called MO means acquiring resources in the external market, which is also similar to Sirmon et al. (2007)'s “acquiring.” Furthermore, this research regards MO and LO as important factors in building global dynamic capabilities, which means that even though the organization uses internal or external relationships to realize resource accumulation and acquisition, it must query and recombine this channel to equip itself with unique marketing ability. The ambidextrous organizational culture of MO and LO emphasizes the involvement and participation of employees, which is aligned with the viewpoint that global dynamic capabilities are achieved by knowledge integration and through the innovative behavior of employees (Meyer et al., 2010; Liu et al., 2019; He et al., 2020 also indicated that the capability cultivation of an organization comes from the accumulation of operation and interaction by knowledge and the psychological characteristics of all employees in the internal process of the organization. Thus, norms formed by a strong culture will strengthen the social network among employees, which is more conducive to improving the efficiency of learning the application and knowledge of new technologies (Peng, 2020). That is, LO shapes the organization's attitude to query and screen resources, which agrees with Sirmon et al. (2007)'s concept of “divesting,” namely that manufacturers should assess the resource and divest the part with less value. Although this research focuses on the correlation between capability, MO, and LO, it also has something to do with resource management. In other words, as the organization establishes resource combination, it can accumulate and acquire resources by MO and use the LO for screening.

This study anaylzed how SMEs utilize cooperation with GPDCs and GMCs in the composition of the governance structure. Our results conform to related literature, which indicates that there is a direct connection between cooperative supplier relations and superior GPDCs and GMCs (Kotabe et al., 2003). SMEs conducting the development of both GPDCs and GMCs are inclined to attach more significance to partnerships in the governance structure. Our method conquers the typical defect in the analysis which relies on the vertical dyadic relationships (Paswan et al., 2017). The results indicate that a portfolio of various relationships is handled strategically and deliberately by SMEs, rather than single ones (Wagner and Johnson, 2004). We stress that GPDCs and GMCs are important in forming SMEs' autonomy and strategic selections when choosing the proper balance between diversified relationships (Helper and Sako, 1995). The results of this study are similar to research by Paswan et al. (2017). In the process of developing GDCs, Internationalized SMEs need to establish a relationship with partners by investing resources, top managers necessarily deal with different situations with different governance structures when making decisions in avoidance of opportunism in inter-firm partnership. Although important, contractual governance is not the exclusive type of governance structure adopted by SMSs. Neglecting other types of governance structure (i.e., relational governance) may be misleading in understanding SMEs' evolution and assessing their competitiveness. Nevertheless, among the different types of capability combinations in the study, except for Low GPDCs/GMCs, all the other capability combinations had a higher preference for relational governance than contractual governance. The possible reason for this is that the selected samples in our study are SMEs that have been internationalized. In the early stages of internationalization, SMEs may be likely to adopt contractual governance when it comes to export or joint venture, but as the operation of the international market tends to be stable, SMEs with mature and international experience have more emphasis on the maintenance and establishment of vertical and horizontal relationships (Dong et al., 2017; Rosenkranz and Wulf, 2019). Thereby, no matter the kind of capability combination, all prefer relational governance. This study also makes an original contribution to the literature on international marketing management. As argued by Mota and de Castro (2005), the majority of studies dealing with relationship portfolios can hardly be used to interpret the coevolution among capabilities and relationships over time.

### Practical Implications

The managerial implications of this study largely relate to how LO and MO are associated with fostering and developing GDCs. MO focuses on processing both foreign market and competitor information, especially on consumers, emphasizing the creation of customer value. If SMEs can establish communication and contact channels with customers, such as through marketing, it can acquire ideas and thoughts on products and augment product functions or customer-preferred products. To collect intelligence from foreign markets and share them in the organization in a more effective manner, SMEs must have sufficient learning mechanisms in place to interpret them, such as learning orientation. This study suggests that managers should build a well-established learning culture, which can provide more understanding and insights from external knowledge and information. Hence, our results also suggest that managers should engage in maintaining and reinforcing MO and LO culture and that they should utilize foreign intelligence processes to drive the development of different capabilities such as GPDCs and GMCs.

Our research states that SMEs tend to choose different governance structures to strengthen their competitiveness in the different development phases of global dynamic capabilities. For internationalized SMEs, although choosing to use contractual governance can be conductive to lowering uncertainty and speculation, the single development and common development of GPDCs and GMCs still stress strengthening interactions with management partners and relational governance as the main direction. This result means that the top managers of SMEs are aware of the vicissitudes and uncertainties in the international market. Close vertical supplier relationships and horizontal enterprise competition and cooperation relationships, established through relational governance. contribute to overcoming various maladaptive situations when enterprises conduct operations overseas. Thus, this study suggests that top managers should first evaluate the capabilities of enterprises, make a suitable adjustment in the resource allocation of capability development, and do a mutual match of governance pattern through the principal development of different capabilities in GDCs rather than complete relational governance or contractual governance.

### Limitations and Future Research

Previous studies on the antecedents of dynamic capabilities have mainly focused on the internal mechanism of an organization; very few have explored its external factors. In addition to the MO and LO discussed in this study, there are also many other important capabilities-building support activities, such as coordination, long-term relationship, and others.

Huge cultural diversities play a vital role in SMEs, as some SMEs included in this study are only based in one country, and the impact of cultural diversity was not considered. This study suggests that GPDCs and GMCs are key components of global dynamic capabilities. Therefore, future researchers are suggested to include SMEs of different countries in their studies to ensure the universality of research results.

In addition, GPDCs and GMCs are different in concept, and there is a tension between them. Due to the factors of theme and context, this study does not further explore this issue, nor does it discuss the possible research gap. Therefore, future studies could focus on this issue, undertaking a more in-depth exploration of whether there are intermediate variables (e.g., information systems between the two, or whether they may be affected by disturbance variables (e.g., environmental situational factors, short-and long-term factors, etc.).

## Data Availability Statement

The original contributions presented in the study are included in the article/Supplementary Material, further inquiries can be directed to the corresponding authors.

## Ethics Statement

The studies involving human participants were reviewed and approved by University of Taipei. The patients/participants provided their written informed consent to participate in this study.

## Author Contributions

This study is a joint work of the two authors. MP contributed to the ideas of research, collection of data, and empirical analysis. MP and GR contributed to the data analysis, design of research methods, and tables. MP and GR participated in developing a research design, writing, and interpreting the analysis. Both authors contributed to the literature review and conclusions.

## Conflict of Interest

The authors declare that the research was conducted in the absence of any commercial or financial relationships that could be construed as a potential conflict of interest.

## References

[B1] Abimbola T. Vallaster C. B. (2007). Organisational identity and reputation in SMEs: on overview. Quali. Market Res. 10, 341–348. 10.1108/13522750710819685

[B2] Andriopoulos C. Lewis M. W. (2009). Managing innovation paradoxes: ambidexterity lessons from leading product design companies. Long Range Plann. 43, 104–122. 10.1016/j.lrp.2009.08.003

[B3] Baker W. E. Sinkula J. M. (1999). The synergistic effect of market orientation and learning orientation. J. Acad. Market. Sci. 27, 411–427. 10.1177/0092070399274002

[B4] Baker W. E. Sinkula J. M. (2002). Market orientation, learning orientation and product innovation: delving inside the organization's black box. J. Market Focused Manage. 5, 5–25. 10.1023/A:1012543911149

[B5] Bitar J. Hafsi T. (2007). Strategizing through the capability lens: sources and outcomes of integration. Manage. Decision 45, 403–419. 10.1108/00251740710745043

[B6] Cadogan J. W. Diamantopoulos A. Siguaw J. A. (2002). Export market-oriented activities: Their antecedents and performance consequences. J. Int. Bus. Stud. 33, 615–626. 10.1057/palgrave.jibs.8491036

[B7] Cantamessa M. (1999). Design best practices, capabilities and performance. J. Eng. Design. 10, 305–328. 10.1080/095448299261227

[B8] Chen C. L. Jaw Y. L. (2009). Building global dynamic capabilities through innovation: a case study of Taiwan's cultural organizations. J. Eng. Tech. Manage. 26, 247–263. 10.1016/j.jengtecman.2009.10.002

[B9] Chen H. L. Hsu W. T. (2009). Family ownership, board independence, and R&D investment. Fam. Bus. Rev. 22, 347–362. 10.1177/0894486509341062

[B10] Chiarvesio M. Di Maria E. Micelli S. (2004). From local networks of SMEs to virtual districts?: evidence from recent trends in Italy. Res. Policy. 33, 1509–1528. 10.1016/j.respol.2004.08.009

[B11] Claro D. P. Hagelaar G. Omta O. (2003). The determinants of relational governance and performance: how to manage business relationships? Industrial Market. Manag. 32, 703–716. 10.1016/j.indmarman.2003.06.010

[B12] Coviello N. Brodie R. Munroe H. (2000). An investigation of marketing practice by firm size. J. Business Venturing 15, 523–545. 10.1016/S0883-9026(98)00035-4

[B13] Dong W. Ma Z. Zhou X. (2017). Relational governance in supplier-buyer relationships: the mediating effects of boundary spanners' interpersonal guanxi in China's B2B market. J. Business Res. 78, 332–340. 10.1016/j.jbusres.2016.12.029

[B14] Dyer J. H. Hatch N. (2006). Relation-specific capabilities and barriers to knowledge transfers: creating advantage through network relationships. Strategic Manage. J. 27, 701–719. 10.1002/smj.543

[B15] Dyer J. H. Singh H. (1998). The relation view: cooperative strategy and sources of interorganization competitive advantage. Acad. Manag. Rev. 23, 660–679. 10.5465/amr.1998.1255632

[B16] Eisenhardt K. Martin J. (2000). Dynamic capability: what are they? Strategic Manag. J. 21, 1105–1121. 10.1002/1097-0266(200010/11)21:10/11andlt;1105::AID-SMJ133andgt;3.0.CO;2-E

[B17] Fang E. Zou S. (2009). Antecedents and consequences of marketing dynamic capabilities in international joint ventures. J. Int. Business Stud. 40, 742–761. 10.1057/jibs.2008.96

[B18] Ferguson R. J. Paulin M. Bergeron J. (2005). Contractual governance, relational governance, and the performance of interfirm service exchanges: the influence of boundary-spanner closeness. J. Acad. Market. Sci. 32, 217–234. 10.1177/0092070304270729

[B19] Furlan A. Grandinetti R. Camuffo A. (2007). How do subcontractors evolve? Int. J. Operat. Production Manage. 27, 69–89. 10.1108/01443570710714547

[B20] Gibson C. B. Birkinshaw J. (2004). The antecedents, consequences, and mediating role of organizational ambidexterity. Acad. Manage. J. 47, 209–226. 10.2307/20159573

[B21] Hair J. Black W. C. Babin B. J. Anderson R. E. (2010). Multivariate Data Analysis, 7th Edn. Upper saddle River, NJ: Pearson Education International.

[B22] Hartigan J. A. Wong M. A. (1979). AK-means clustering algorithm. J. R. Stat. Society. 28, 100–108. 10.2307/2346830

[B23] He H. Bai Y. Gao J. Xie J. (2020). How RandD staff's improvisation capability is formed: a perspective of micro-foundations. Front. Psychol. 11:2337. 10.3389/fpsyg.2020.551970 33013596 PMC7509410

[B24] Helper S. Sako M. (1995). Supplier relations in Japan and the United States: are they converging? Sloan Manage. Rev. 36, 77–84.

[B25] Ho Y. C. Tsai T. H. (2006). The impact of dynamic capabilities with market orientation and resource-based approaches on NPD project performance. J. Am. Acad. Business 8, 215–228.

[B26] Hooley G. Greenly G. Cadogan J. Fahy J. (2005). The performance impact of marketing resources. J. Business Res. 58, 18–27. 10.1016/S0148-2963(03)00109-7

[B27] Hsu L. C. Wang C. H. (2012). Clarifying the effect of intellectual capital on performance: the mediating role of dynamic capability. Br. J. Manag. 23, 179–205. 10.1111/j.1467-8551.2010.00718.x

[B28] Hult G. T. M. Ketchen S. S. F. (2005). Market orientation and performance: an integration of disparate approaches. Strategic Manage. J. 26, 1173–1181. 10.1002/smj.494

[B29] Jantunen A. PuumalaÕnen K. Saarenketo S. KylaheÕko K. (2005). Entrepreneurial orientation, dynamic capabilities and international performance. J. Int. Entrepreneurship 3, 223–243. 10.1007/s10843-005-1133-2

[B30] Kaynak H. (2003). The relationship between total quality management practices and their effects on firm performance. J. Oper. Manag. 21, 405–435. 10.1016/S0272-6963(03)00004-4

[B31] Knight G. A. Cavusgil S. T. (2004). Innovation, organizational capabilities, and the born-global firm. J. Int. Business Stud. 35, 124–141. 10.1057/palgrave.jibs.8400071

[B32] Kogut B. Zander U. (1992). Knowledge of the firm, combinative capabilities, and the replication of technology. Organizat. Sci. 3, 383–397. 10.1287/orsc.3.3.383

[B33] Kohli A. K. Jaworski B. J. (1990). Market orientation: the construct, research propositions, and managerial implications. J. Market. 54, 1–18. 10.1177/002224299005400201

[B34] Kohli A. K. Jaworski B. J. Kumar A. (1993). MARKOR: a measurement of market orientation. J. Market. Res. 30, 467–477. 10.1177/002224379303000406

[B35] Kotabe M. Martin X. Domoto H. (2003). Gaining from vertical partnerships: Knowledge transfer, relationship duration, and supplier performance improvement in the U.S. and Japanese automotive industries. Strategic Manage. J. 24, 293–316. 10.1002/smj.297

[B36] Liu Y. Wang W. Chen D. (2019). Linking ambidextrous organizational culture to innovative behavior: a moderated mediation model of psychological empowerment and transformational leadership. Front. Psychol. 10:2192. 10.3389/fpsyg.2019.02192 31681063 PMC6798063

[B37] Liu Z. (2005). Reading behavior in the digital environment: changes in reading behavior over the past ten years. J. Doc. 61, 700–712. 10.1108/00220410510632040

[B38] Lumpkin G. T. Lichtenstein B. B. (2005). The role of organizational learning in the opportunity recognition process. Entrepreneurship Theory Pract. 29, 451–472. 10.1111/j.1540-6520.2005.00093.x

[B39] March J. (1991). Exploration and exploitation in organizational learning. Organizat. Sci. 2, 71–87. 10.1287/orsc.2.1.71

[B40] Martin X. Salomon R. (2003). Knowledge transfer capacity and its implications for the theory of the multinational corporation. J. Int. Business Stud. 34, 356–373. 10.1057/palgrave.jibs.8400037

[B41] Menguc B. Auh S. (2008). The asymmetric moderating role of market orientation on the ambidexterity-firm performance relationship for prospectors and defenders. Ind. Market. Manag. 37, 455–470. 10.1016/j.indmarman.2007.05.002

[B42] Meyer J. P. Hecht T. D. Gill H. Toplonytsky L. (2010). Person–organization (culture) fit and employee commitment under conditions of organizational change: a longitudinal study. J. Vocational Behav. 76, 458–473. 10.1016/j.jvb.2010.01.001

[B43] Miner A. S. Bassof P. Moorman C. (2001). Organizational improvisation and learning: a field study. Admin. Sci. Q. 46, 304–337. 10.2307/2667089

[B44] Morgan N. A. Vorhies D. W. Mason C. (2009). Market orientation, marketing capabilities, and firm performance. Strategic Manage. J. 30, 909–920. 10.1002/smj.764

[B45] Morgan N. A. Zou S. Vorhies D. W. Katsikeas C. S. (2003). Experiential and informational knowledge, architectural marketing capabilities, and the adaptive performance of export ventures. Decision Sci. 34, 287–321. 10.1111/1540-5915.02375

[B46] Mota J. de Castro L. M. (2005). Relationship portfolios and capability development: cases from the moulds industry. J. Purchasing Supply Manage. 11, 42–54. 10.1016/j.pursup.2005.04.002

[B47] Narver J. C. Slater S. F. (1990). The effect of a market orientation on business profitability. J. Market. 54, 20–35. 10.1177/002224299005400403

[B48] O'Cass A. Weerawardena J. (2010). The effects of perceived industry competitive intensity and marketing-related capabilities: drivers of superior brand performance. Industrial Market. Manage. 39, 571–581. 10.1016/j.indmarman.2009.04.002

[B49] O'Dwyer M. Gilmore A. Carson D. (2009). Innovative marketing in SMEs: an empirical study. J. Strategic Market. 17, 383–396. 10.1080/09652540903216221

[B50] Özsomer A. Simonin B. L. (2004). Marketing program standardization: a cross-country exploration. Int. J. Res. Market. 21, 397–419. 10.1016/j.ijresmar.2004.06.003

[B51] Paswan A. K. Hirunyawipada T. Iyer P. (2017). Opportunism, governance structure and relational norms: an interactive perspective. J. Business Res. 77, 131–139. 10.1016/j.jbusres.2017.04.012

[B52] Peng M. Y. P. (2020). Ambidexterity in social capital, dynamic capability and SMEs' performance: quadratic effect of dynamic capability and moderating role of market orientation. Front. Psychol. 11:4024. 10.3389/fpsyg.2020.584969 33613352 PMC7892958

[B53] Peng M. Y. P. Lin K. H. (2019). International networking in dynamic internationalization capability: the moderating role of absorptive capacity. Total Qual. Manage. Business Excellence 1–20. 10.1080/14783363.2019.1661239

[B54] Peng M. Y. P. Zhang Z. Yen H. Y. Yang S. M. (2019). Dynamic capabilities and firm performance in the high-tech industry: quadratic and moderating effects under differing ambidexterity levels. Sustainability 11:5004. 10.3390/su11185004

[B55] Peng Y. P. Lin K. H. (2017). The effect of global dynamic capabilities on internationalizing SMEs performance: the culture factors as antecedents. Baltic J. Manage. 12, 307–328. 10.1108/BJM-09-2016-0199

[B56] Pinho J. C. Prange C. (2016). The effect of social networks and dynamic internationalization capabilities on international performance. J. World Bus. 51, 391–403. 10.1016/j.jwb.2015.08.001

[B57] Ploetner O. Ehret M. (2006). From relationships to partnerships: new forms of cooperation between buyer and seller. Industrial Market. Manage. 35, 4–9. 10.1016/j.indmarman.2005.08.006

[B58] Podsakoff P. M. Organ D. W. (1986). Self-reports in organizational research: Problems and prospects. J. Manag. 12, 531–544. 10.1177/0149206386012004088452065

[B59] Prange C. Verdier S. (2011). Dynamic capabilities, internationalization processes and performance. J. World Bus. 46, 126–133. 10.1016/j.jwb.2010.05.024

[B60] Rindova V. P. Petkova A. P. (2007). When is a new thing a good thing? Technological change, product form design, and perceptions of value for product innovations. Org. Sci. 18, 217–232. 10.1287/orsc.1060.0233

[B61] Rosenkranz C. Wulf T. (2019). Behavioral integration as a relational governance mechanism in family firms—The moderating role of family involvement in management. J. Small Business Manage. 57, 801–819. 10.1111/jsbm.12325

[B62] Schreyögg G. Kliesch-Eberl M. (2007). How dynamic can organizational capabilities be? Towards a dual-process model of capability dynamization. Strategic Manage. J. 28, 913–933. 10.1002/smj.613

[B63] Sidhu J. S. Commandeur H. R. Volberda H. W. (2007). The multifaced nature of exploration and exploitation: value of supply, demand, and spatial search for innovation. Organizat. Sci. 18, 20–38. 10.1287/orsc.1060.0212

[B64] Sinkula J. Baker W. Noordewier T. (1997). A framework for market-based organizational learning: linking values, knowledge, and behaviour. J. Acad. Market. Sci. 25, 305–318. 10.1177/0092070397254003

[B65] Sirmon D. G. Hitt M. A. Ireland R. D. (2007). Managing firm resources in dynamic environments to create value: looking inside the black box. Acad. Manage. Rev. 32, 273–292. 10.5465/amr.2007.23466005

[B66] Slater S. F. Narver J. C. (1995). Market orientation and the learning organization. J. Market. 59, 63–74. 10.1177/002224299505900306

[B67] Slater S. F. Narver J. C. (1998). Customer?led and market?oriented: let's not confuse the two. Strat. Manag. J. 19, 1001–1006. 10.1002/(SICI)1097-0266(199810)19:10<1001::AID-SMJ996>3.0.CO;2-4

[B68] Swaminathan V. Moorman C. (2009). Marketing alliances, firm networks and firm value creation. J. Market. 73, 52–69. 10.1509/jmkg.73.5.52

[B69] Swan K. S. Kotabe M. Allred B. B. (2005). Exploring robust design capabilities, their role in creating global products, and their relationship to firm performance. J. Product Innovation Manage. 22, 144–164. 10.1111/j.0737-6782.2005.00111.x

[B70] Teece D. J. (2007). Explicating dynamic capabilities: the nature and micro-foundations of (Sustainable) enterprise performance. Strategic Manage. J. 28, 1319–1350. 10.1002/smj.640

[B71] Teece D. J. Pisano G. Shuen A. (1997). Dynamic capabilities and strategic management. Strategic Manage. J. 18, 509–533. 10.1002/(SICI)1097-0266(199708)18:7andlt;509::AID-SMJ882andgt;3.0.CO;2-Z

[B72] Tushman M. L. O'Reilly C. A. I. I.I. (1996). Ambidextrous organizations: managing evolutionary and revolutionary Change. California Manage. Rev. 38, 8–30. 10.2307/41165852

[B73] Vahlne J. E. Jonsson A. (2017). Ambidexterity as a dynamic capability in the globalization of the multinational business enterprise (MBE): case studies of AB Volvo and IKEA. Int. Business Rev. 26, 57–70. 10.1016/j.ibusrev.2016.05.006

[B74] Vorhies D. W. Morgan N. A. (2005). Benchmarking marketing capabilities for sustained competitive advantage. J. Market. 69, 80–94. 10.1509/jmkg.69.1.80.55505

[B75] Wagner S. M. Johnson J. L. (2004). Configuring and managing strategic supplier portfolios. Industrial Marketing Manage. 33, 717–730. 10.1016/j.indmarman.2004.01.005

[B76] Wang C. L. Ahmed P. K. (2007). Dynamic capabilities: a review and research agenda. Int. J. Manage. Rev. 9, 31–51. 10.1111/j.1468-2370.2007.00201.x

[B77] Winter S. G. (2003). Understanding dynamic capabilities. Strat. Manag. J. 24, 991–995. 10.1002/smj.318

[B78] Zahra S. A. Sapienza H. J. Davidsson P. (2006). Entrepreneurship and dynamic capabilities: a review, model and research agenda. J. Manag. Stud. 43, 917–955. 10.1111/j.1467-6486.2006.00616.x

[B79] Zhang M. Tansuhaj P. (2007). Organizational culture, information technology capability, and performance: the case of born global firms. Multinational Business Rev. 5, 43–79. 10.1108/1525383X200700012

[B80] Zhang W. Chen R. R. (2013). Dynamic capability and IJV performance: the effect of exploitation and exploration capabilities. Asia Pacific J. Manage. 30, 601–632. 10.1007/s10490-010-9235-3

[B81] Zollo M. Winter S. G. (2002). Deliberate learning and the evolution of dynamic capabilities. Organizat. Sci. 13, 339–351. 10.1287/orsc.13.3.339.2780

