# Corrigendum: The Impact of Global Dynamic Capabilities on Governance Structure Choice of Partnership: The Moderating Effect of Ambidexterity

**DOI:** 10.3389/fpsyg.2021.679604

**Published:** 2021-10-05

**Authors:** Guoying Ren, Michael Yao-Ping Peng

**Affiliations:** ^1^Business School, Beijing Normal University, Beijing, China; ^2^School of Economics & Management, Foshan University, Foshan, China

**Keywords:** global dynamic capabilities, Governance structure, learning orientation, market orientation, international marketing

In the original article, there was an error in the authorship as published. After a unanimous decision, the authors consider that Din Jong had a low contribution to this article and is not qualified to be an author of this article. Thus, the author Din Jong has been removed from the author list.

The authors apologize for this error and state that this does not change the scientific conclusions of the article in any way. The original article has been updated.

## Publisher's Note

All claims expressed in this article are solely those of the authors and do not necessarily represent those of their affiliated organizations, or those of the publisher, the editors and the reviewers. Any product that may be evaluated in this article, or claim that may be made by its manufacturer, is not guaranteed or endorsed by the publisher.

